# Association of Brachial-Ankle Pulse Wave Velocity and Cardiomegaly With Aortic Arch Calcification in Patients on Hemodialysis: Erratum

**DOI:** 10.1097/01.md.0000491160.92211.9e

**Published:** 2016-08-12

In the article “Association of Brachial-Ankle Pulse Wave Velocity and Cardiomegaly With Aortic Arch Calcification in Patients on Hemodialysis”, ^1^ which appeared in Volume 95, Issue 19 of *Medicine*, Dr. Shih's name was originally spelled incorrectly. The article has since been corrected online.

## References

[1] Shin M-CP Lee M-Y Huang J-C Association of brachial-ankle pulse wave velocity and cardiomegaly with aortic arch calcification in patients on hemodialysis. *Medicine.* 2016 95:e3643. 27175684 10.1097/MD.0000000000003643 PMC4902526

